# Teaching Anatomy to Neuroscientific Health-Care Professionals: Are They Receiving the Best Anatomical Education?

**DOI:** 10.1007/s40670-019-00838-7

**Published:** 2019-11-06

**Authors:** Francesco Latini, Mats Ryttlefors

**Affiliations:** Department of Neuroscience, Neurosurgery, Uppsala University, S-751 85 Uppsala, Sweden

**Keywords:** Medical education, Lab demonstration, Brain anatomy, White matter, Teaching anatomy, Neurosurgical training

## Abstract

University neuroanatomical courses seldom teach the anatomical-functional connectivity of the brain. White matter dissection improves understanding of brain connectivity, but until now has been restricted to neurosurgeons and in some cases to medical students, never to health-care non-medical professionals. Our aim was to teach white matter anatomy to medical and non-medical students to evaluate this technique in groups with different education. A standardized lab demonstration of white matter anatomy was performed with high appreciation rate in both groups, suggesting a suboptimal neuroanatomical education provided by basic course. We encourage to include this technique of teaching brain anatomy into basic neuroanatomical courses to improve the level of comprehension and competence in all health-care staff within the field of neuroscience.

## Introduction

Knowledge of superficial and deep brain anatomy provides a better comprehension of clinical symptoms and surgical or medical treatment of brain pathologies. Basic courses on brain anatomy seldom include modern theories on white matter organization and correlated brain functions [1–3]. Advances in medical imaging, including the use of digital devices and virtual reality, provide new opportunities for learning brain anatomy, but lab demonstration and dissection is still fundamental to a solid learning experience during the basic courses [4–9].

In most university programs worldwide where lab demonstrations of brain anatomy are performed, the classical approach for studying deep cerebral architecture is based on cadaver dissection and two-dimensional (2D) projections/slices of brain specimens [4, 8]. This method is problematic in identifying 3D anatomy, especially origins and terminations of all white matter bundles [8, 10].

Better learning results can be achieved with white matter fiber dissection, a technique based on blunt dissection of white matter pathways removing the cortex of formalin-fixed brains [10–14]. White matter dissection has substantially contributed to our knowledge on brain connectivity [8, 11–15]. All the neuroscientific health-care professionals are interested in learning these aspects of brain anatomy and functions, but until now, white matter dissection has mostly been utilized by neurosurgeons and in some cases in teaching medical students.

Nurses, nurse assistants, physiotherapists, and other health-care professionals are significantly involved during their professional life in taking care of patients with complex brain lesions at the onset, perioperatively, or during the rehabilitative process. All these categories are not systematically included in advanced courses for learning brain anatomy which resulted in lack of comprehension of anatomical-functional-clinical implications of brain lesions. A more detailed education on brain anatomy and functional connectivity can facilitate comprehension of the neurological/neurosurgical disease and even the rationale for a specific treatment (i.e., awake surgery for tumors harbored in eloquent areas) or the potential mechanism of neuroplasticity and rehabilitation.

Few articles in the literature clearly encourage the use of this technique in teaching white matter anatomy to undergraduate students [8, 9, 13], while no previous article suggests the use of this technique for teaching purposes to non-medical students (in this context, non-medical refers to undergraduate students in nursing, physiotherapy, and other professionals in health care and students in basic neuroscience).

In this study, we report the application of a standardized white matter dissection demonstration to undergraduate medical and non-medical students to improve their understanding of brain anatomy and 3D orientation of brain architecture and its clinical/surgical implications.

## Activity

A total number of 40–60 medical students (20–30 per group every semester) received lectures and lab session on deep brain anatomy per year. The same standardized demonstration was also performed for nurse students (40 per year), physiotherapists (10–20 per year), and non-medical staff of neurosurgical and neurological department at Uppsala University Hospital (30 per year).

**Fig. 1:**
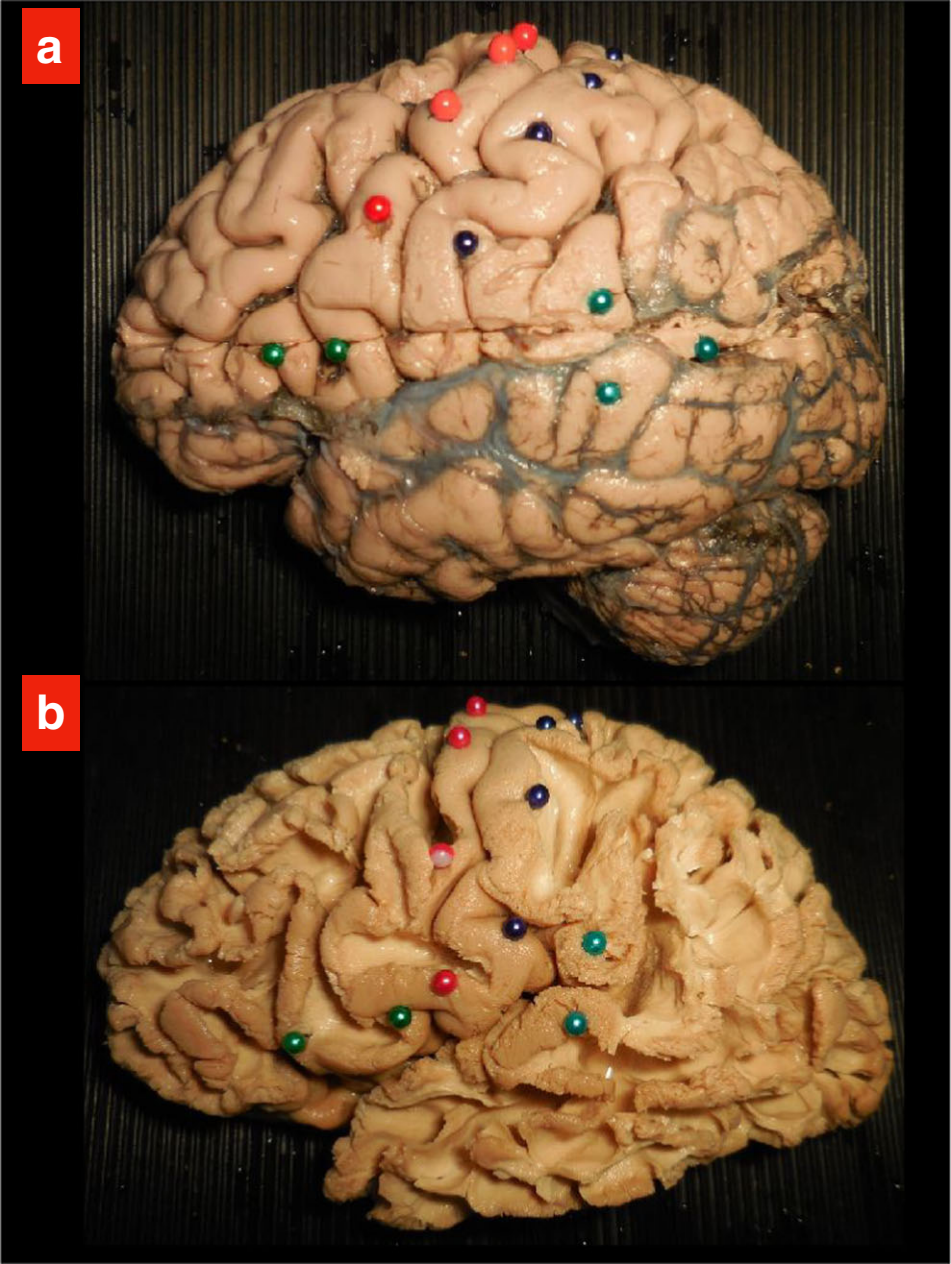
The picture illustrates the first two exemplary steps of white matter dissection. In **a**, the initial specimen is utilized to show superficial/gyral dissection and the major lobes. In the upper part of the specimen, the arachnoid layer and the cortical vessels have been removed to better illustrate the anatomical/surgical relationship between cortex and deep meningeal layer. Colored pins are used during the dissection to facilitate orientation from the cortical level to the midline structures (in this case the Broca’s area, precentral and postcentral gyrus and Wernicke’s area are marked). **b** A second step of the dissection when most of the cortex within the sulci has been removed and the first layer of subcortical connectivity including short U-fibers is exposed

**Fig. 2:**
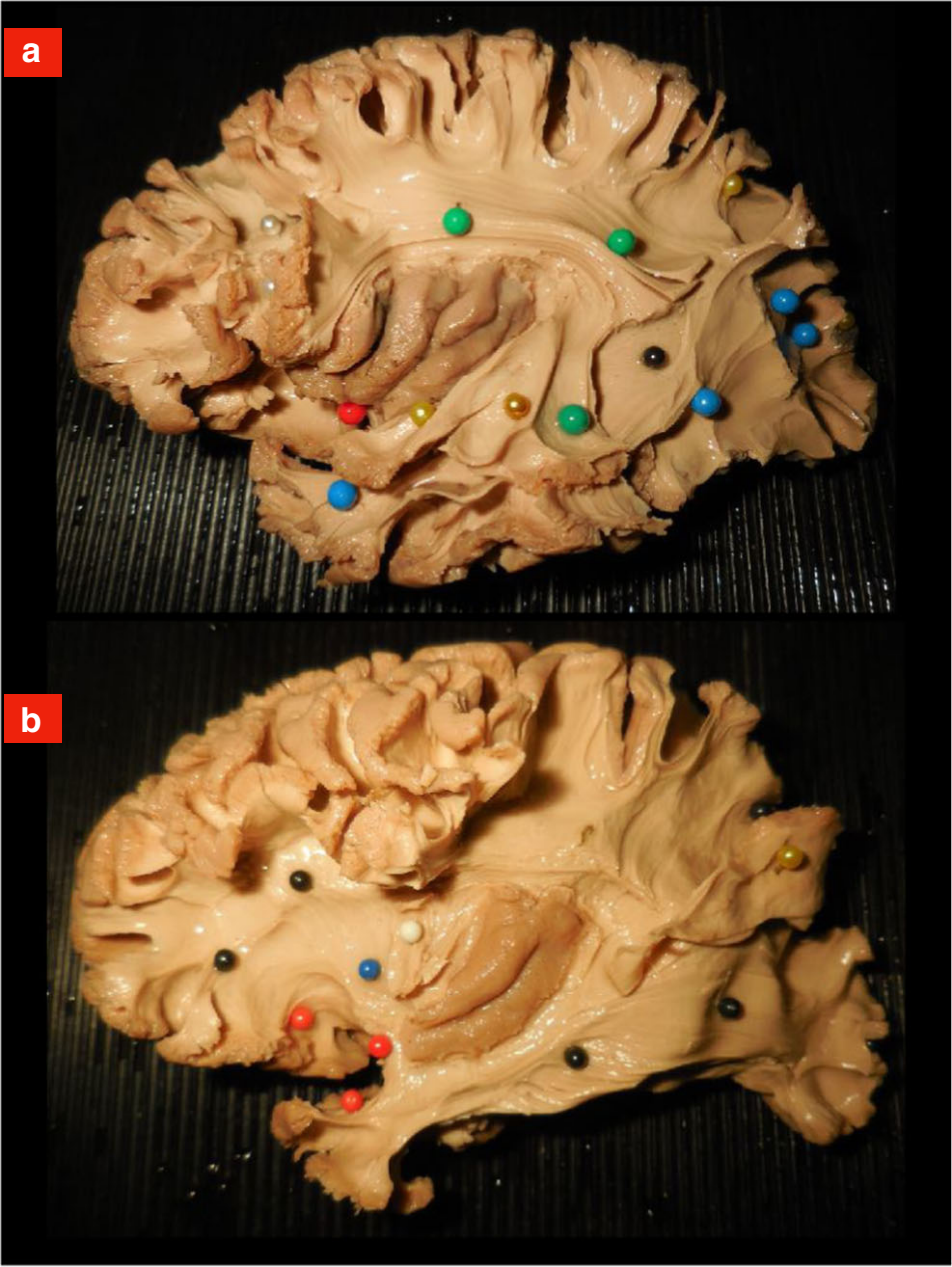
Exemplary specimens used during the demonstration. **a** The central sulcus region has been removed revealing the whole course of the arcuate fasciculus (green pins). On the temporo-occipital region, the whole course of the two major longitudinal fibers systems (inferior longitudinal fasciculus, blue pins; and middle longitudinal fasciculus, yellow pins) has been exposed. Functional interaction between the systems as well as information provided by direct cortical/subcortical brain mapping are described to facilitate comprehension of the anatomy. Relationships with the insular lobe are illustrated, introducing the uncinate fasciculus (red pin) and the aslant tract (white pins). **b** A deeper layer of white matter is illustrated retracting the middle longitudinal fasciculus (yellow pin) and revealing the inferior fronto-occipital fasciculus (black pins) within the sagittal stratum of Sachs. Anteriorly in fronto-temporal region, the course of the uncinate fasciculus is now exposed from the lateral view (red pins). Only the posterior portion of the insula (long gyri) has been saved to illustrate the anatomy of the extreme capsule (white pin) and the external capsule (dark blue pin). Three-dimensional orientation as well as function supported by these structures are provided during the demonstration

A standardized stepwise dissection of the white matter architecture was demonstrated through 10 previously dissected specimens (Figs. 1 and 2) to emphasize the surgical-anatomical point of view for students with no previous understanding of neurosurgical brain anatomy. The specimens were used for education as a part of a larger research project on white matter dissection as previously described in other articles [16–19].

The students were divided in groups of 4–8 persons. The instructor presented an oral introductory overview about the theory of white matter organization, functional connectivity of brain functions, and neuroplasticity in the initial part of the lab demonstration. The whole demonstration took 45 to 60 min depending on the subsequent discussion and questions.

An anonymous evaluation questionnaire was administered to all the students at the end of each course and filled out after the demonstration. The medical students were asked to evaluate the learning activity (lab demonstration) on a scale from 1 to 5 (in progressive order of appreciation), where 1 stands for useless/insufficient and 5 stands for excellent. Non-medical students were asked to evaluate the learning activity on a scale of 1 to 10 where 1 stands for useless/insufficient experience and 10 stands for very important/positive experience. All the students were also asked to comment in free text their thoughts and experiences of the learning activity and/or suggest improvements. The differences in the scales reflected the same appreciation scoring methods that each group was familiar with during the other educational courses.

## Results and Discussion

**Fig. 3:**
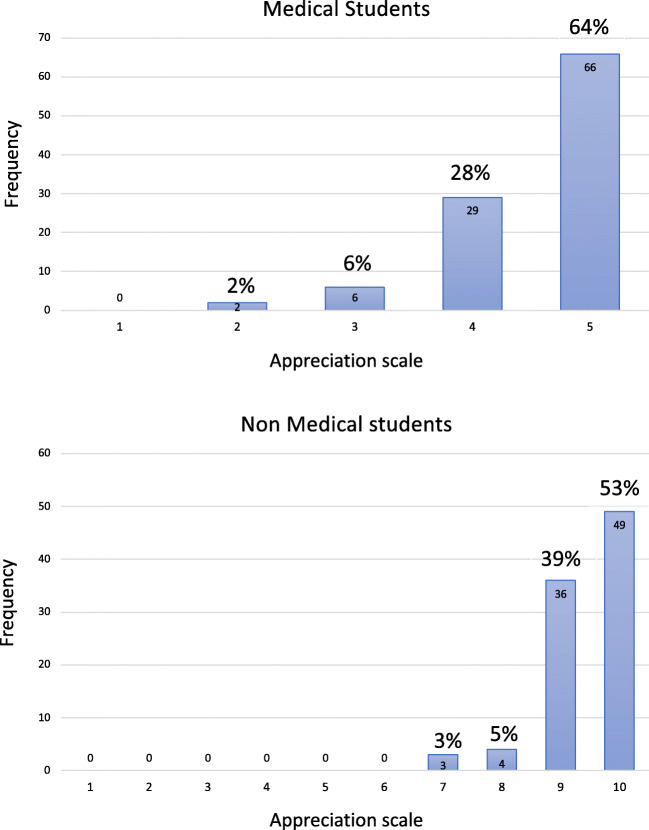
The bar graphs show the quantitative results from the questionnaire analysis. In the upper part is the frequency histogram for medical students showing the distribution of the appreciation scores. In the lower part is the frequency histogram for non-medical students showing the distribution of the appreciation scores. The quantitative score from the questionnaires was analyzed using RStudio (Version 1.1.456–© 2009-2018 RStudio, Inc.)

A total number of 201 students attended the white matter dissection demonstrations during the last 2.5 years and 97% of them returned the questionnaire. The general reception of the demonstration was very positive for all the groups. The evaluation rates for medical students (103 questionnaires; IQ1 4.0, median 5.0, mean 4.5, IQ3 5.0) and non-medical students (92 questionnaires; IQ1 7.0, median 10.0, mean 9.4, IQ3 10.0) are displayed in Fig. 3. Most of the comments from medical students described the experience as an important and illustrative/didactic complement to their previous knowledge of 2-dimensional anatomy. For para-medical participants, the comments described how interesting and stimulating it was for them to see the 3D anatomy, instead of studying anatomy in a book and/or computer-based programs. The common suggested improvement was the need to include the white matter anatomy learning activity into the basic course for anatomy and brain functions.

Our experience in teaching brain anatomy with the support of white matter dissection demonstrated assuring results in terms of feedback and usefulness by the participants.

The aim of the demonstration was to give the students a visual 3D experience on deep white matter architecture. The possibility to handle the specimens with exposed trajectories and cortical terminations was considered as very helpful in understanding the real anatomy underneath the cortex. Ninety-two percent of the medical students considered the lab demonstration an excellent or very important experience in learning brain anatomy (Fig. 3). This is in accordance with many articles supporting the importance of physical demonstration rather than computer-based anatomy for learning improvement [4, 8, 9]. According to their knowledge based on standard anatomical lectures and lab demonstrations on brain anatomy, they could not name more than two structures involving white matter, i.e., corpus callosum and internal capsule. Moreover, when asked, the real orientation of these two structures was poorly understood. The lack of anatomical knowledge for a medical doctor is a potential problem in understanding the clinical implications of a brain damage and its related treatment.

This is a very important and well-established method in neurosurgical training [11, 13]. Contributing to a better understanding of the structural connectivity of the brain [11, 13, 20] and due to the good consistency with modern techniques of neuroimaging [20, 21], the use of white matter dissection in neurosurgical and neuroradiological training has been widely encouraged [11, 22]. However, we believe that not only neurosurgeons should be able to understand deep brain anatomy but also any medical student should receive a comprehensive education on three-dimensional brain anatomy during their basic medical education. Future general practitioners, neurologists, psychiatrist, psychologists, and rehabilitation specialists play key roles within the neuroscientific field. A better understanding of brain anatomy can positively influence their skills and understanding of brain diseases.

Our results demonstrated another important implication of this teaching process and modality. Nurses, nurse assistants, physiotherapists, and other professionals are very interested in learning brain anatomy at an advanced level. Ninety-two percent of the non-medical students judged this experience as excellent or very important for anatomy learning (Fig. 3).

Our results support two critical considerations. First, despite the differences in general level of education, both medical students and other health-care professionals are receiving an incomplete neuroanatomical education. Second, other categories of health professionals, previously excluded from advanced neuroanatomical courses, are interested in learning and understanding more about brain anatomy and its functional connectivity.

Hence, we strongly suggest to systematically include this technique into basic neuroanatomical courses to medical students, non-medical students, and other health-care professionals. This would improve the level of comprehension and competence in all health-care staff within the field of neuroscience. Future studies with quantitative and qualitative comparisons between teaching techniques will be necessary to confirm how the clinical and rehabilitative aspects could be improved by a better anatomical knowledge.
